# Evaluation of addictive behavior in depressive disorders

**DOI:** 10.1192/j.eurpsy.2023.1363

**Published:** 2023-07-19

**Authors:** A. Tounsi, Z. Bencharfa, H. Chebli, M. Sabir, F. El Omari

**Affiliations:** 1 Psychiatric; 2 Addiction, Arrazi university psychiatric hospital, Sale, Morocco

## Abstract

**Introduction:**

The comorbidity between depressive disorders and addiction is far from being random. Through substances, users try to ameliorate their feelings of sadness, reduce present anxiety. The phenomena of tolerance and dependence quickly worsen the situation, and make any attempt at withdrawal more difficult.

**Objectives:**

The objective of this study is to analyze the addictive behavior in patients diagnosed with depressive disorder.

**Methods:**

This is a retrospective descriptive study carried out by analyzing hospitalization records in the addictology department of the psychiatric university hospital Ar-Razi in Salé over a period of one year (from August 2020 to August 2021). The diagnoses are established according to the DSM 5 diagnostic criteria.

**Results:**

Of 141 patient records initially entered, nine records were not usable and 54 patients had a diagnosis of depressive disorder constituting 40.9% of admissions to the service. The average age was 37.9 years (16; 69).

Among our depressed and substance-using patients, the most frequent comorbidity was personality disorders (29.6%) followed by anxiety disorders (11.1% of cases). Thirty-five percent of patients reported at least one suicide attempt in the past and 11.1% had experienced sexual abuse.

The average age of onset of addiction in our sample was 17.8 years (11; 31). The most used substance was tobacco (n=44) followed by alcohol (n=43), cannabis and then benzodiazepines.

**Conclusions:**

The relationship between depression and substance use remains complex. Although depressed patients often turn to drugs in search of a state of well-being, withdrawal from these substances can also aggravate or cause the depression.

**Disclosure of Interest:**

None Declared

